# Predictive factors and risk and protection groups for loneliness in older adults: a population-based study

**DOI:** 10.1186/s40359-024-01708-7

**Published:** 2024-04-26

**Authors:** Pedro Montejo Carrasco, David Prada Crespo, Ana Isabel Reinoso García, Monserrat Lozano Ibáñez, Borja Montejo Rubio, Mercedes Montenegro-Peña

**Affiliations:** 1 Centre for the Prevention of Cognitive Impairment, Madrid Salud, Madrid, Spain; 2 Department of Experimental Psychology, Faculty of Psychology, Complutense University of Madrid (UCM), Madrid, Spain; 3 Ramón y Cajal University Hospital of Madrid, Madrid, Spain; 4 Department of Basic Psychology I, Faculty of Psychology, National University of Distance Education of Madrid, Madrid, Spain

**Keywords:** Ageing, Depression, Living alone, Loneliness, Mental health, Protective factors, Quality of life, Risk factors

## Abstract

**Background:**

Loneliness is considered a public health problem, particularly among older adults. Although risk factors for loneliness have been studied extensively, fewer studies have focused on the protected and risk groups that these factors configure. Our objective is to analyze the variables and latent factors that predict loneliness in older adults and that enable risk and protected groups to be configured.

**Methods:**

We employed an epidemiological, cross-sectional survey that was carried out on a random sample of 2060 people over 65 years extracted from the census. A structured telephone interview was used to assess mental and physical health, habits, quality of life, and loneliness, applying the COOP-Wonca, Goldberg General Health (GHQ-12), and Barber Questionnaires.

**Results:**

Predictors of loneliness were: mental health, living alone, quality of life, depressive symptoms, low educational level, and some deficiency situations such as having no one to turn to for help. The factors extracted (Factorial Analysis) were: a subjective experience of poor health, objective isolation, and psychological isolation. We established at risk and protected groups (“Decision Tree” procedure), and loneliness was referred to by 73.2% of the people living alone and with poor mental health and quality of life (risk group). By contrast, only 0.8% of people living with others, with good mental health and good quality of life felt loneliness (protected group).

**Conclusion:**

In a well-developed city, subjective and objective factors are associated with loneliness. These factors, especially those associated with at risk or protected groups, must be considered to develop strategies that address loneliness.

## Introduction

Loneliness is of increasing concern in developed countries. Although clinicians and social scientists expressed interest in this issue many decades ago [1], it has only recently been recognised as a public health problem [2]. As early as 1968, loneliness, social isolation, and living alone were identified as related but independent situations [3]. Now, it is clear that the construct of loneliness is complex, with researchers attributing it to two fundamental dimensions: emotional, being subjective, and social, being objective [4].

In the emotional, subjective dimension, the focus is the absence of “close emotional attachment.” There is a lack of meaningful relationships or ties with the environment [5] and almost always a lack of emotional support [6]. We can consider two components in the emotional aspect: the cognitive and the affective. The cognitive one is the perception, recognition, and awareness of the “absence” of attachment. The affective is the feeling and the experience of this absence; it is accompanied by various emotions that are usually primarily negative, such as sadness, uneasiness, and mistrust. However, they can also be positive, such as calmness or many other emotions. These two types of conflicting emotions can even occur simultaneously in an often ambivalent process, depending on the individual or the situation. The social dimension is objective and implies relational factors. There is a lack of social network. Some authors consider that the social objective dimension presents at least two clearly differentiated areas with different characteristics: the connection with individuals close to one (spouse-partner…), sometimes referred to as “intimate others”, and the extensive social networks established [7–9]. Social dimension can be associated with a certain marginality outside any group [10].

These dimensions are not mutually exclusive; they overlap, together forming a multidimensional construct [11]. Considering the two faces of loneliness is crucial when studying the causal and triggering factors, the “at-risk” and “protective” groups, as well as measuring and analysing its limits.

The frequency of loneliness for older adults varies across countries. The pooled estimates for developed countries were 7.9% for people reporting severe loneliness and 25.9% for moderate loneliness [12]. In Europe: Northern European countries had the lowest pooled prevalence, 5.2%, followed by Western Europe at 8.7%, Southern Europe at 15.7% and Eastern European countries at 21.3%. Pooled prevalence data for Europe and USA is 13% [13]. In Spain, the prevalence is 9.2% [14].

The literature reveals the enormous complexity of the potential causal or triggering factors of loneliness in older individuals, which can be categorized as follows: Sociodemographic aspects like age or way of life (living alone or with a partner) [15]; health factors like chronic disease or compromised mobility [16]; resources, personal links or social support networks and imposed deficiencies like dependence [17]; life events such as deaths or moving home [18]; cultural and structural factors like means of interaction [19]; type and place of housing, meeting places [17, 20], etc. Moreover, loneliness has also been associated with certain lifestyles or compromised situations, such as when no one cares about you, having no one to turn to if you need help, or not setting aside time for self-care [21]. In addition, there are associations of loneliness with multiple health disorders [22] including cardiovascular diseases like hypertension, immune, sleep disorders [23], depression and anxiety [24], suicide [25], as well as cognitive impairment and even dementia [26, 27]. Loneliness is also a risk factor associated with mortality [28].

A crucial point is the methodology used to study groups of individuals at higher risk of loneliness. Risk groups have been derived in part from what we have called the causes of loneliness, whereas elsewhere risk groups were also established through Latent Class Analysis [29]. Moreover, groups or typologies have been formed based on the feeling of loneliness, social isolation and living alone. Living alone has been considered one of the most critical risk factors for loneliness [30], although this is not always the case [31]. Elsewhere Cross-relationship Panel Analysis [32] has been used to study the prospective associations between loneliness and depression. The regression methods employed were usual, not only for cross-sectional studies but also, to analyze the longitudinal effects of certain risk factors [33] like physical and mental health, chronic disease, anxiety symptoms or environmental factors like social cohesion, in conjunction with risk markers like age and gender. However, we believe there are few studies that have analyzed and established at risk and, in particular, protected groups. Regarding loneliness assessment, validated scales, as well as direct questions like “Have you felt lonely?” for population studies, are commonly used [15, 34].

Here we used the “Madrid Salud 2018” survey, a cross-sectional study that considers epidemiological, social, health, disability, and lifestyle variables. Our first objective was to analyze the predictors of feeling loneliness in older people. Our hypothesis was that living alone would be the strongest predictor of loneliness. In addition, poor quality of life, depressive symptoms, suffering certain deficiencies (having no one to turn to in case of needing help, feeling that no one cares about you, not eating a hot meal in the last few days, lacking basic social support measures, or lower educational level) and health problems that are to some extent disabling, including sensory problems (major visual or hearing deficiencies) or diseases that affect daily living (e.g., osteoarthritis) would also be predictors. Our second objective was to establish groups that are at risk and those that are protective based on the aforementioned variables. Our hypothesis was that the risk groups would comprise older people who live alone, have mental health problems, experience some deficiency, or suffer sensory alterations or limiting diseases. Alternatively, loneliness would be infrequent in those individuals who live with others, who have a good quality of life, solid mental health and who are not suffering from any deficiency or diseases that affect their social interactions, such as hearing or visual impairment. Although risk factors for loneliness have been studied extensively, fewer studies have focused on the protective groups. Therefore, we intended to add to this issue by identifying characteristics or variables that may be associated with these protected and risk groups. The third objective was to analyze the possible latent factors underlying the different predictors of loneliness. The hypothesis was that these variables would group as objective and subjective factors that together would explain the loneliness construct. From these latent factors obtained, we intended to offer an explanatory, albeit partial, model of loneliness development.

Our main contribution is the study of the risk and the protective factors in groups within a specific urban population. We believe that this is another way to study the predictors of loneliness, grouping them as they occur in reality in a large population, and enabling a probability to be established that an individual influenced by such factors of risk and protection will experience loneliness.

## Methods

### Ethics

The Carlos III Health Institute Ethics Committee approved the survey applied and its protocols (No. CEI PI 51-2017-v2). Participants were informed that the data was confidential and anonymized. Consent was obtained verbally at the beginning of the interview, after having read to the selected person a text explaining the interview (origin, purpose, duration…) in which the data protection law was mentioned. The person was interviewed if he/she gave his/her consent.

### Procedure

The data presented here was generated in the “Madrid Salud 2018 Survey”, a transversal and descriptive study [35]. Based on this census, stratified random sampling was designed considering districts, age, and sex. The survey was carried out by telephone using a structured questionnaire and by professionals trained in epidemiological studies. The survey was administered by interviewers who worked at a company dedicated to conducting surveys, and who were trained specifically for this task by Madrid Salud and the company. The data was collected by computer-assisted telephone interview, using the BELVIEW (6.12f) computer system. This is a real-time computer-assisted telephone interviewing system based on a structured questionnaire following the CATI30 approach [36]. The system supports applications that send and receive calls, and that can be connected to external databases.

The interview lasted 35–45 min, selecting the interviewees randomly based on their age, sex and location, and selecting cell phones and landlines in equal proportion. The database of cell phones was used as a database of randomly generated numbers. The questionnaire was administered to whoever answered the phone if they met the age, sex and district requirements. Calls were not repeated and nor were people substituted for others in the household. In households connected by landline telephone, the person to be interviewed was selected randomly from those who met the criteria of the search and the completed groups (age, sex and district).

### Inclusion and exclusion criteria

To be at least 15 years of age, living in Madrid, and to be able to answer the questions (e.g., comprehension, language). For those who could not answer (e.g., deafness, failure to understand the questions, inability to speak the language or cognitive impairment), a proxy was used. The questions the proxy could answer and those they could not had been established previously, avoiding those that referred to personal issues difficult for another person to respond to. For these people any unanswered questions are left blank.

### Measurements

This questionnaire addressed socio-demographic, health, social, and lifestyle habits, and as frequently performed in epidemiological studies, questions were asked about illnesses (e.g., “Has your doctor told you that you suffer from an illness…?“) [37], treatments, services and assistance, disability, sleep, etc. Several instruments have been used in the Survey: COOP-Wonca, GHQ, Barber Questionnaire and the MMSE orientation for time items. The psychometric properties of all of them were analyzed for the Spanish population.

We employed the 12-item Goldberg General Health Questionnaire (GHQ-12) [38, 39] that screens for non-psychotic mental pathologies (depression, anxiety, stress, sleep…) and includes items like loss of sleep over worry, constantly feeling under strain, able to enjoy day-to-day activities, feeling unhappy and depressed. It is a recommended questionnaire for health surveys [40]. GHQ can be used as a Likert Scale with 0–4 options (range 0–48) and a cut-off point of 3/4 [41], indicating probable case/no case (Likert score). When it is used as a screening for mental pathology (GHQ score), it has a sensitivity of 83.4% and a specificity of 76.3% [38]. The instrument’s internal consistency for Spanish population has a Cronbach’s alpha of 0.86 for general population and 0.90 for people over 65 years old. In this sample, Cronbach’s alpha is 0.792; Hotelling’s T square, F = 475.53, *p* < 0.001; Spearman-Brown coefficient is 0.813. In the factor analysis performed by Rocha et al. [41] (KMO: Likert score 0.91 and GHQ score 0.93), the variance explained by a single factor is 67% for Likert score and 73% for GHQ score so it can be used as a one-dimensional scale. Others authors have highlighted the GHQ multidimensionality. Regarding its validity, GHQ-12 screening gave a positive result for 62% of the respondents who reported having suffered from depression, anxiety or any other mental health disorder, indicating the possible presence of a mental disorder. Similarly, a significant association was observed between self-perceived health status and GHQ-12 (significant χ2, *p* < 0.001) and 64% percent of those who stated that their health status was fair, poor or very poor, gave a positive response in the GHQ-12 [41].

The COOP-Wonca Questionnaire [42] measures health-related QoL, including 9 items focusing on physical fitness, daily activities, social activities, feelings, changes in health, and overall health. It has a 5-point response scale, with 1 meaning the best way of functioning and 5, the worst (range 9–45). The psychometric characteristics for its administration by telephone were studied in the population in Madrid (Spain) using a sample drawn randomly from the City’s census. Regarding the psychometric characteristics of the questionnaire the internal consistency is Cronbach’s alpha 0.93, and the factor analysis finds a single factor that explains 78.8% of the variance. In terms of validity, the correlation with the global perception of the patient’s health over the past 12 months was 0.70 [43].

The Barber Fragility Questionnaire [44] has 9 items of interest, including: “Are there days when you cannot have a hot meal? Do you have difficulty with your vision?… or hearing? Are you without a relative you could call on for help?” It has a sensitivity of 95% and a specificity of 68% to discriminate for functional risk [44]. The psychometric parameters have been studied and validated for telephone interviews with the target population of this survey. In the factor analysis performed in this population (Bartlett = 1,270.3; *p* < 0.001; KMO is 0.71), the variance explained for a single factor is 45.3%; the reliability Cronbach`s alfa was 0.76 [43]. The correlation between the Barber Questionnaire with health-related quality of life (QoL) was R^2^ = 0.26 and, with loneliness, was R^2^ = 0.17. Other studies have correctly differentiated older people with greater or lesser frailty [45, 46]. In these three questionnaires, a higher score indicates mental health problems, poorer QoL, and greater frailty.

In addition, we asked several questions about memory complaints and time orientation, the latter taken from the Mini-Mental State Examination [47]. The validation of the MMSE for the Spanish population has been carried out by several authors on different population types [48, 49]. Blesa et al. [50], studying control volunteers and patients with mild cognitive impairment and dementia, found a sensitivity of 82.0 and a specificity of 92.9. Regarding its reliability, Cronbach’s alfa was 0.94 and the test-retest reliability was 0.87. Time orientation items have been used as a cognitive test in some studies and correlates very closely with the MMSE total score [51]. Other authors found that time orientation is the only domain that predicts subsequent cognitive decline in older people [52]. For time orientation items, Lobo et al. [53] employing the cut-off point ¾, found a sensitivity of 81.3%, a specificity of 91.5% and area under ROC Curve of 0.915.

### Loneliness questions

Three specific questions were used to assess loneliness: Have you felt lonely in the last year? Do you live alone? How many people live with you? The possible answers to this first question were: always or nearly always; often; rarely; or never/very rarely. We distributed these answers into two categories: “feeling lonely” (the first two responses) and “not feeling lonely” (the last two). These questions probe two fundamental contents of the loneliness construct: the feeling of loneliness and social isolation. Using the above questions allows us to encompass both the subjective cognitive-affective and the objective elements of loneliness [30]. Although these phenomena can be analyzed independently, it is crucial to study them in conjunction due to their close relationship. The first question is the fundamental one of the survey. It addresses the discrepancy between the quantity/quality of the available social relationships and those desired or needed by the individual.

### Statistical analysis

The SPSS Statistics software was used (IBM SPSS Statistics for Windows, Version 20.0. IBM Corp.): The dependent variable was loneliness or feeling lonely. The independent variables were studied by organizing them into areas of related questions. Categorical variables were coded such that the higher number always indicated a larger load or pathology. ANOVA was used to study the association between the dependent and independent variables. The effect size was assessed through the Eta^2^ (Eta^2^ 0.01 = small effect, 0.06 = medium effect, 0.14 = large effect) [54] and contingency tables with the “Cramer’s V” statistic (0.07/0.10 = small, 0.21/0.30 = medium, 0.35/0.50 = large, for 2/1 degrees of freedom) [54].

To study predictors, we used a logistic regression stepwise method, with the Odds Ratio (OR) and its level of confidence (CI), and Nagelkerke’s R^2^ (a correction of the Cox and Snell R^2^). The estimated R^2^ was that proposed by Cohen: 0.02 ‘small’, 0.13 ‘medium’, and 0.26 ‘large’ [49]. We first performed regression studies with each one of the blocks of variables, and as possible predictor variables, we used all those that were seen to be significant in this study and that had a Cramer’s V effect size > 0.10. The groups and variables introduced were: (1) Sociodemographic variables - age, sex, living alone, and educational level; (2) Cognitive performance - memory problems that affect daily living; (3) Mental health - depressive symptomatology, general mental health, and taking tranquilizers; (4) Disease and QoL - QoL (COOP-Wonca) and perception of health status, visual difficulties, and hearing impairment; pain variables - general pain (yes/no), arthritis/arthrosis; (5) Deficiencies - having health problems that prevent one from looking after themselves, feeling that no one is concerned about them, having no one to turn to for help, and not eating hot meals more than twice a week; (6) Life habits - taking time to care for oneself and feel good. To further reduce the variables that predict loneliness and to obtain the latent factors, we conducted a factorial analysis (principal component analysis -PCA) of the statistically significant predictive factors identified in the regression analysis. We used the VIF statistic to analyze multicollinearity in regression studies, which was estimated according to the criteria of Montgomery, Peck, and Vining [55]. The coefficients associated with the regression equation are thought not to be correctly estimated at VIF values above 5 due to multicollinearity.

We used the SPSS “Decision Tree” procedure to define the “at risk” and “protected” groups, and we included the predictor variables obtained in the logistic regression. We followed two strategies: (1) considering the whole population as a single group and establishing the risk and protected groups using the predictor variables; (2) dividing the sample based on the variable with the highest OR, which divides the population into two categories (e.g., living alone - yes/no), an objective variable that is easy to measure and that can split the population into those who lived alone and those who did not. Given that we studied the risk groups based on the variable living alone in this second strategy, we dispensed this variable in the first strategy. We divided the General Mental Health variable (GHQ-12) into 3 categories: good mental health, 1 (1 point in GHQ-12); poor mental health, 4 (4 or more points in GHQ-12); and intermediate status, 2 or 3 (2 or 3 points in GHQ-12). The tree procedure divided the variable QoL (COOP-Wonca) into ≤ 23 points and > 23 points. In the decision trees presented, the dependent variable was feeling lonely. In the risk group trees, each node displays the number and percentage of people who feel lonely (= Yes) and those who do not feel lonely (= No); the last line presents the total of each node and the percentage of the entire sample represented by the individuals in that node. The corresponding “p” of statistical significance and the corresponding test Chi^2^ is also shown before each partition. The Barber Questionnaire was not analyzed as only one global score but with different scores corresponding to each item in order to highlight the deficient situations. It contains items such as: Are there any days when you are unable to have a hot meal? Are you without a relative you could call on for help? Do you depend on someone for regular help? Most questions were administered to the entire sample. However, some participants did not offer responses for all the variables, and in such cases, we removed these participants from the specific analysis.

## Results

Table 1 presents the socio-demographic data and the percentage of loneliness. We observed that 9.2% of the entire sample of older people were lonely. While 27.5% live alone, of these, 19.7% feel lonely, whereas 5.3% of the sample feel lonely despite living with others. We performed bivariate associations with epidemiological, social, health, and lifestyle variables related to loneliness (Table 2). We also observed stronger associations with depressive symptoms, living alone, general mental health, and QoL (COOP-Wonca). The size of the effect of the committed circumstances variable should be considered.

**Table 1:** Socio-demographic characteristics of the sample, number of participants, percentage of the total sample, and percentage of feeling lonely

Characteristics: *N* = 2060; age range: 65–98 years; mean age: 73.26 (SD: 6.19)			
Variable	N	%	% Feeling lonely
**Age groups**			
65–69	703	34.1	7.1
70–74	528	25.6	7.2
75–79	565	27.4	11.9
80–84	153	7.4	11.8
85 and over	111	5.5	15.3
**Sex**			
Male	795	38.6	11.5
Female	1265	61.4	5.7
**Social work class**			
Manager/College	726	35.2	6.2
Skilled worker	752	36.5	9.7
Semi-/unskilled worker	529	25.7	12.3
Has not worked	53	2.6	12.2
**Level of studies**			
Primary or less	594	28.8	15.1
Secondary	822	39.9	7.7
University students (medium/high)	644	31.3	5.1

**Table 2:** Association among dependent (loneliness) and independent variables

Variables	Bivariate study		
	Statistic	(p) sig.	Effect size
1. **Sociodemographic Variables**			
Age	Chi^2^ = 65.90	< 0.0001	V = 0.18
Sex	Chi^2^ = 80.39	< 0.0001	V = 0.20
Level of studies	Chi^2^ = 46.17	< 0.001	V = 0.15
Living alone	Chi^2^ = 100.99	< 0.001	V = 0.22
Social work class	Chi^2^ = 15.10	< 0.002	V = 0.09
2. **Cognitive Performance**			
Memory problems affect your daily life	Chi^2^ = 6.84	0.009	V = 0.19
Have you seen a doctor about this?	Chi^2^ = 1.05	0.305	V = 0.07
Memory complaints	Chi^2^ = 13.30	< 0.001	V = 0.11
Temporal Orientation	F = 6.83	< 0.01	Eta^2^ = 0.007
3. **Mental Health**			
General Mental Health (GHQ 0–12)	F = 452.03	< 0.001	Eta^2^ = 0.18
Depression	Chi^2^ = 167.38	< 0.001	V = 0.29
Chronic Anxiety	Chi^2^ = 91.98	< 0.001	V = 0.21
Quality of sleep	Chi^2^ = 22.09	< 0.001	V = 0.15
4. **Quality of life health related and Disease**			
Quality of life health related (COOP-Wonca)	F = 285.20	< 0.001	Eta^2^ = 0.12
Multimorbidity (0–11)	Chi^2^ = 29.81	< 0.001	V = 0.12
Difficulties in seeing	Chi^2^ = 45.56	< 0.001	V = 0.15
Difficulties in hearing	Chi^2^ = 18.68	< 0.001	V = 0.10
Pain (general)	Chi^2^ = 66.78	< 0.001	V = 0.18
Taking opioids (last two weeks)	Chi^2^ = 26.60	< 0.001	V = 0.11
Arthritis/arthrosis	Chi^2^ = 48.76	< 0.001	V = 0.15
High cholesterol	Chi^2^ = 3.24	0.072	V = 0.04
Hypertension	Chi^2^ = 9.08	0.003	V = 0.07
5. **Deficiency situations**			
Has health problems that prevent him/her from taking care of him/herself	Chi^2^ = 11.27	< 0.001	V = 0.23
Feel that no one is concerned about one’s self	Chi^2^ = 45.94	< 0.001	V = 0.15
Having no one to turn to for help	Chi^2^ = 58.45	< 0.001	V = 0.17
Do not eat hot food more than two days a week	Chi^2^ = 42.53	< 0.001	V = 0.14
Barber Questionnaire	F = 253.66	< 0.001	V = 0.11
6. **Life Habits**			
Physical activity you can do	Chi^2^ = 87.73	< 0.001	V = 0.21
Take time to care of oneself and feel good	Chi^2^ = 37.18	< 0.001	V = 0.14
Frequency of alcohol consumption	Chi^2^ = 17.91	< 0.001	V = 0.09
Smoking	Chi^2^ = 3.99	0.262	V = 0.04
Having someone who cares about the older person	Chi^2^ = 64.02	< 0.001	V = 0.18
Having animals at home (pets)	Chi^2^ = 0.53	0.467	V = 0.02

(p) sig.: statistical significance

Due to the relevance of these two variables (age and sex), we analyzed them in particular. Table 1 shows the data on loneliness of men and women (5.5% versus 11.5%). Depressive symptoms were found in 5.5% of men and 14.8% of women (*p* < 0.0001; V Cramer: 0.14). The Mental Health Score (GHQ) is higher (indicating worse mental health) in women than in men (*p* < 0.0001; R^2^: 0.018). Living alone 16.4% of the men in the sample and 34.5% of the women (*p* < 0.0001; V Cramer: 0.20). The perception of quality of life (COOP-Wonca) is lower in women (*p* < 0.0001; R^2^: 0.047). Regarding age, the correlation between QoL (COOP-Wonca) and age is *r* = 0.206 (*p* < 0.0001) and with Mental Health is *r* = 0.089 (*p* < 0.0001). If we look at the relationship between living alone and age, we see that older people live more frequently alone (*p* < 0.0001; R^2^:0.032) and are slightly more depressed (*p* < 0.006; R^2^: 0.004).

### Predictors (logistic regression)

Table 3 shows the predictor variables according to the final model, defining their OR and the confidence interval (CI) (Table 3). The final model was significant (Chi^2^ = 412.05 *p* < 0.0001; log-likelihood = 846,919), and the model correctly predicted 92.3% of the participants (98.5% of those feeling lonely and 30.7% of those not feeling lonely; R^2^ Nagelkerke’s = 0.396). We found that no variable had a VIF above 5 (Table 3) when analyzing multicollinearity in the regression studies. We concluded that the variables in the model are not co-linear (i.e.: they are independent in the regression). Then, the standard errors for the coefficients were correctly estimated. Some variables were not entered into the equation: pain, taking tranquilizers, the general perception of health (COOP-Wonca), visual and hearing difficulties, arthritis/arthrosis, and spending time caring for oneself.

**Table 3:** Predictive variables. Logistic regression. Dependent variable: loneliness

Variables	Wald	Sig.	OR	95% CI Inf-Sup	VIF
Sex	0.136	0.712	0.923	0.601–1.415	1.112
Age	0.767	0.381	1.013	0.984–1.042	1.112
Living alone	58.977	0.000	4.506	3.069–6.617	1.100
General Mental Health (GHQ)	44.080	0.000	1.307	1.208–1.414	1.695
Quality of life (COOP-Wonca)	14.410	0.000	1.085	1.040–1.131	1.722
No one is concerned about the older person	15.666	0.000	2.238	1.502–3.336	1.035
Depression	13.009	0.000	2.279	1.457–3.567	1.261
Do not eat hot food more than two days a week	10.173	0.001	2.933	1.514–5.681	1.027
Having no one to turn to for help	6.857	0.009	1.968	1.186–3.265	1.055
Primary (or less) Education Level	5.100	0.024	1.335	1.039–1.716	1.100
Constant	81.628	0.000			

OR: Odds Ratio; 95% C.I.: 95% Confidence Interval; sig.: statistical significance; VIF: Variance Inflation Factor.

We could see that the predictor variable with the greatest effect was living alone (Table 3). Other significant variables in the predictive model were some situations of deficiency (“no one is concerned about the older person”, “having no one to turn to for help”, “not eating hot meals more than twice a week”). In addition, other predictor variables were poor perceived mental health, depressive symptoms (one of the main mental health disorders), low QoL and a low educational level.

### Latent variables (factor analysis)

There were three blocks or groupings of variables in the factor analysis, explaining 50.3% of the variance (KMO = 0.625, Bartlett Chi^2^ = 965.87, *p* < 0.0001: Table 4). The first group involved comprised QoL, general mental health, depressive symptoms, and primary education. The second group included living alone, “having no one to turn to for help,” and “do not eat hot food more than twice a week”, while the third group contained the variable “no one is concerned about the older person”.

**Table 4:** Factor analysis

Predictive factors	Components		
	1	2	3
Quality of Life (COOP-Wonca)	0.814		
General Mental Health (GHQ)	0.777		
Depression	0.598		
Primary Education Level	0.377		
Having no one to turn to for help		0.703	
Living alone		0.543	
Do not eat hot meal more than two days a week		0.468	
No one is concerned about the older person			0.878

### Risk groups

**Fig. 1:**
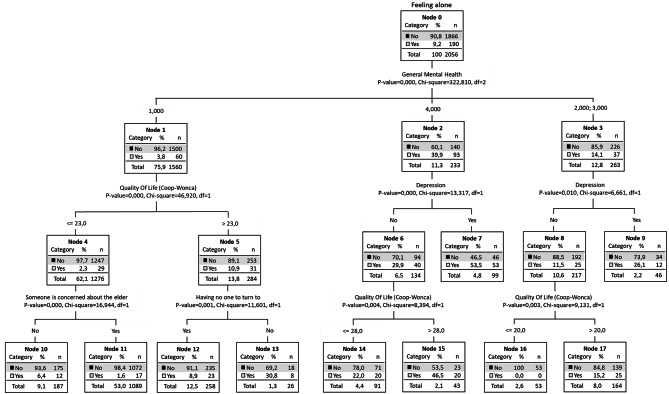
Decision tree - Risk groups with the entire sample (*n* = 2056)

**Fig. 2:**
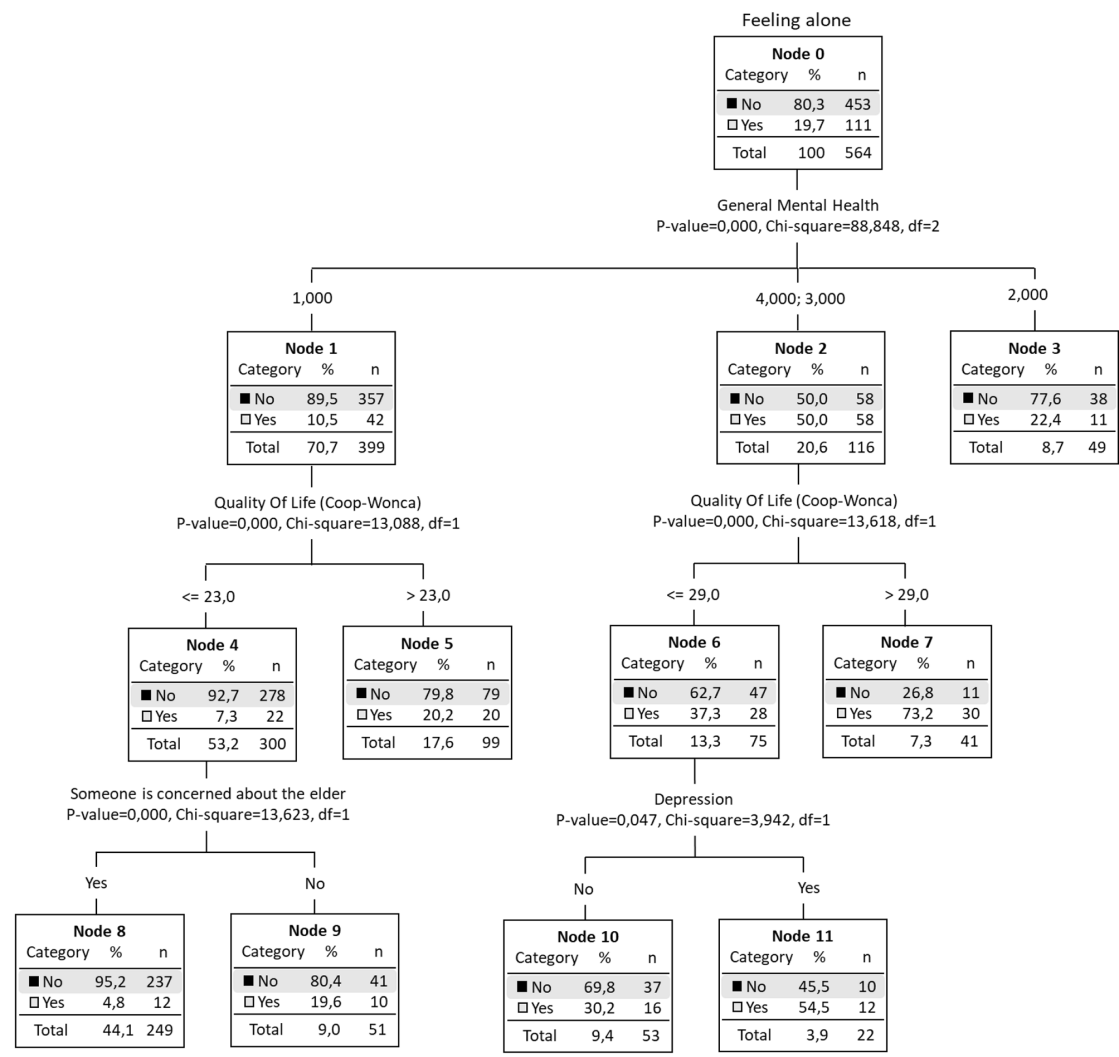
Decision tree. Risk groups with individuals living alone (*n* = 564)

**Fig. 3:**
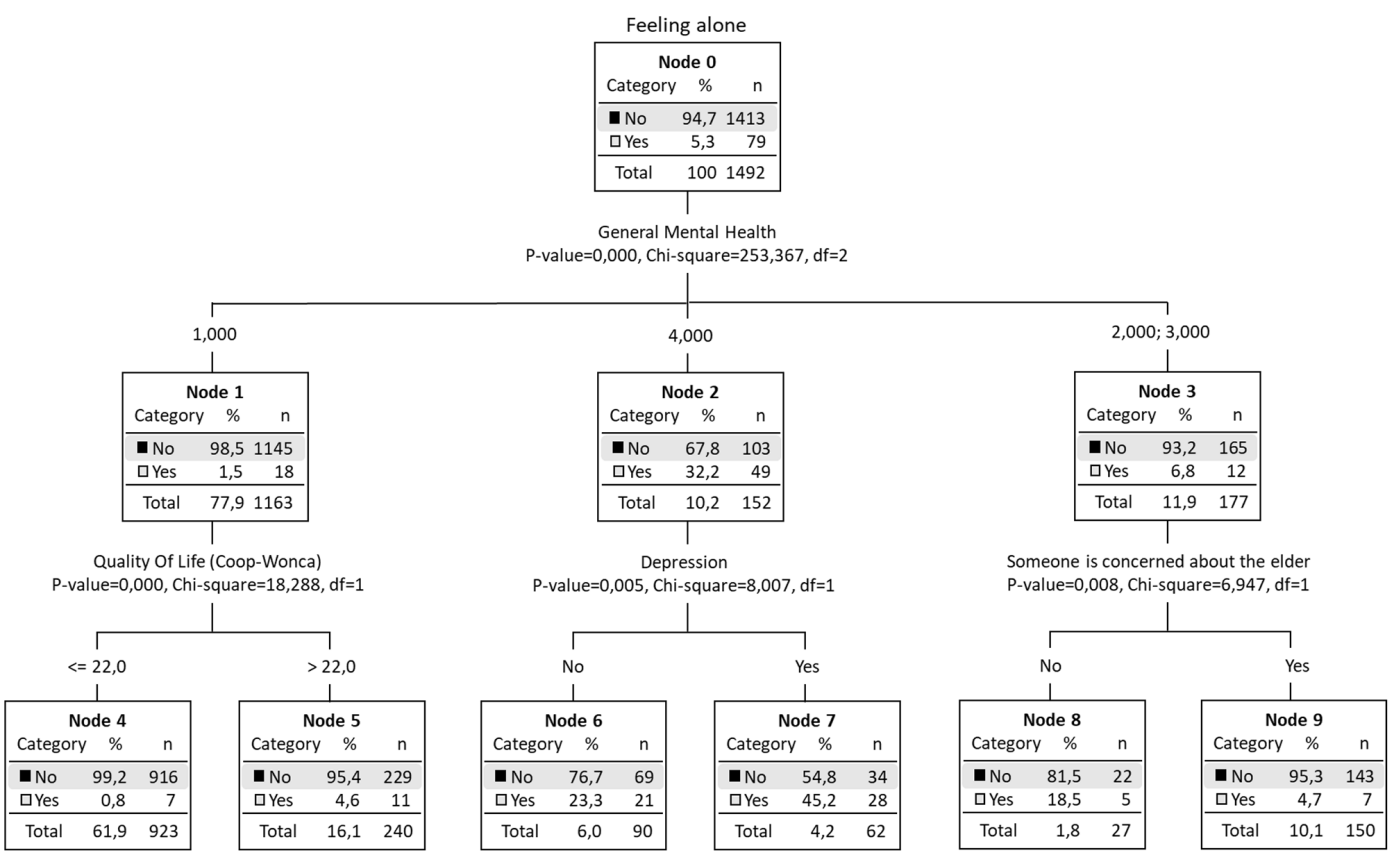
Decision tree. Risk groups with individuals who do not live alone (*n* = 1492)

The “at-risk” and “protected” groups were established from the entire population (Figs. 1, 2 and 3). In Fig. 1, node 0 shows the overall prevalence of loneliness (9.2%), and the first division is made with the general mental health variable. The risk groups, that is, those with the highest percentage of people who feel lonely, were found in nodes 2 (39.9% feel lonely), 6 (29.9%), 7 (53.5%), 13 (30.8%) and 15 (46.5%). The variables that contribute to these were having mental health problems, poor QoL, suffering from depressive symptomatology and having no one to turn to for help. By contrast, the groups that were protected were found in node 1 (3.8% feel lonely), node 4 (2.3%), 11 (1.6%), and 16 (0%), and the variables that contributed to these were: having good mental health, good QoL, someone is concerned about the older person, and not suffering from depressive symptoms.

We also found that an average of 19.7% of people “feeling lonely” were found in the group of people living alone (node 0) (Fig. 2), and the principal variable involved was general mental health as measured with the GHQ. The risk groups with the highest proportion of people who feel lonely were evident in nodes 2 (50% of the subjects are lonely), 6 (37.3%), 7 (73.2%; in this node were people who were living alone, with mental health problems and a poor QoL), 10 (30%) and 11 (54.5%). Finally, there was a protected group, node 8, in which only 4.8% of people felt lonely (living alone but with good mental health, good QoL, and someone who is concerned about them).

For people who do not live alone, we see that the percentage of those who feel lonely was 5.3% (Node 0), which is significantly lower than those who lived alone, as also observed in the rest of the percentages analyzed globally (Fig. 3). As seen previously, general mental health was the first condition dividing this group. Indeed, the risk group with the highest proportion of people who feel lonely was represented in node 2, in the group with mental health problems (32.2% feel lonely), and in node 7; the percentage of loneliness rises to 45.2%, where there were also depressive symptoms among those that reported mental health problems. The protected groups were represented in node 1 (individuals living with others who are in good mental health) with only 1.5% of loneliness (18 individuals out of 1,163), and in node 4 (0.8% feel lonely), 5 (4.6%) and 9 (4.7%). As in the group of those living alone, the variables that contributed to these groups were mental health, QoL, depressive symptoms, and having someone who is concerned about the older adult.

## Discussion

We conducted a randomized cross-sectional epidemiological study based on the census of a large European city. From the data obtained, we examine the predictors, the latent variables, and the “at risk” and “protected” groups for loneliness.

Regarding our first objective, we found that living alone is the predictor of loneliness with the largest effect size. Other significant variables in the predictive model were poor perceived mental health, depressive symptoms (one of the main mental health disorders), QoL, some situations of deficiency (“no one is concerned about the older person”, “having no one to turn to for help”, “not eating hot meals more than twice a week”) and a low educational level. The data obtained partially confirmed our first hypothesis and indicated that other disabilities or health problems, such as deficient sight or hearing, are not predictors of loneliness.

Our second objective was to establish risk and protective groups. We found risk factors that can define groups of people in which a high proportion of them feel lonely. For example, more than half of those individuals with mental health problems, depressive symptomatology, or who live in deficient circumstances feel lonely. The group in which the highest proportion of individuals are considered lonely (73.2% feeling lonely) are those who live alone, with poor mental health and a worse QoL. In terms of protective factors, we identified groups in which the fewest number of individuals experience loneliness. Among people who do not live alone, that have good mental health and a higher QoL, less than 1% feel lonely. Therefore, the initial hypothesis we postulated is confirmed.

Concerning our third objective, in accordance with the hypothesis raised, we identified three latent factors, two that correspond to the subjective and objective components of loneliness. The third is a novel finding, indicating that a lonely person’s consideration of their health, QoL, and psychosocial handicap may be valid predictors of that condition.

### Sociodemographic characteristics: age and sex

The bivariate study made it evident that loneliness is more prominent in older people and women. Although numerous studies confirm this relationship with gender [15], when analyzed with other variables like living alone or the average lifespan of men and women, this association may be called into question [56]. For example, a study only detected a higher frequency of loneliness in older women (≥ 85 years) [31], while a questionnaire-based online study of 46,956 participants aged between 16 and 99 found loneliness was more common in men than in women [57]. Importantly, several studies found a higher proportion of loneliness in young people [14, 58], and as such, a U-shaped curve with a higher frequency of loneliness in younger and older individuals has been proposed [57]. In our study, loneliness was more frequent in older adults.

In line with the above, the association of age and sex with loneliness found in our bivariate study should be interpreted based on the relationship of both these variables with the main predictors (living alone, depressive symptoms, mental health, and QoL). This interpretation was confirmed in the multivariate analysis, in which age and sex lost significance in the predictive model. Consequently, the influence of these sociodemographic parameters on loneliness is probably not an independent effect, but rather, it depends on the other variables considered. It is likely that any group with features similar to those associated with age and sex will experience more loneliness.

### Living alone

Living alone is a risk factor for loneliness with a strong effect size, and according to our data, one-in-five people living alone feel lonely. In establishing the risk groups, we divided them based on this variable, which helped to identify target populations. In our society, older people often live alone as they are unmarried or widowed, the latter a situation complicated by the mourning and loss of the deceased partner, which could be a trigger for loneliness. Being widowed has long been studied as a source of loneliness [4] and the protective effect of having a partner is consistently defined in cross-national studies [15]. Moreover, associations have been found between living alone and various physical conditions, including mental health problems, depression, a worse QoL and a higher risk of mortality [2, 59, 60]. In our study, while loneliness was twice as prevalent in those who live alone relative to the whole population, it is essential to note that 80% of those living alone do not feel lonely. Loneliness and social isolation (e.g., living alone) are discrepant constructs that are usually only weakly correlated [61]. Thus, there are several nuances that must be considered when contemplating the common notion that “living alone” or “social isolation” lead to significant health problems.

When studying discrepancies between loneliness/isolation, the concepts of susceptibility and resistance to loneliness arise. The idea of “social asymmetry” has been proposed to describe these discrepancies, defining four categories with two concordant (“Low loneliness with Low isolation” and “High loneliness with High isolation”) and two discordant groups (“High loneliness with Low isolation” and “Low loneliness with High isolation”). These four groups have different susceptibilities or resistance to physical and mental disorders [62], with better cognitive performance in the “Low loneliness and High isolation” than in the other groups. In a 7-year follow-up population-based study using the same social asymmetry metric, the discordant groups and the “High loneliness with High isolation” group were associated with a higher risk of mortality from different causes [63]. Other findings support this data, and in a longitudinal study of 7,032 older adults, those who lived alone and did not feel lonely were less at risk of depressive disorders, chronicity (2 or more chronic diseases), impaired activities of daily living or poor health status [29]. By contrast, those who lived alone and felt lonely were more likely to suffer from such health problems. Some older people live alone and are at greater risk of suffering disorders in mental health and a worse QoL, whereas others cope better with this situation and manage their solitude adequately, with no adverse risk to their health, state of mind or daily activities. In short, the different elderly individual’s resilience will have a decisive influence on the objective consequences of isolation, as seen in recent studies on COVID related isolation [64].

### Mental health

The association of mental health with loneliness has been well established, and our results indicate that most individuals with mental health problems who suffer from depressive symptoms also suffer from loneliness. In a study on the prevalence and risk factors of loneliness in the UK, six independent factors related to vulnerability and mental health were identified (as studied with GHQ-12), as well as depression [65]. It was subsequently found that loneliness is the strongest predictor of depression in older people, with an OR of 17.76 (15.96–19.76), as is the persistence of depression with an OR = 5.93 (CI: 5.54–6.34) [66]. Based on these findings, it could be argued that there is a bidirectional relationship between loneliness and mental health. However, in a longitudinal study, changes in loneliness predicted changes in depressive symptomatology but not vice versa [32]. Despite the controversies, we consider the relationship is probably bi-directional to at least some extent, such that poor mental health can lead to loneliness through symptoms like anhedonia or a weaker desire for contact with the outside world. In fact, loneliness may be a symptom of moderate depression (despite its non-inclusion in the DSM-5). In turn, loneliness conditions poor mental health, low self-esteem, negative interpretations of relationships with others (e.g., feeling rejected) and feelings of incompetence [67], and it increases the risk of depressive symptoms [32, 66].

### Risk and protective groups

The study of the risk factors of loneliness at times seems to have overshadowed that of protective factors [68]. However, we must consider that many of these factors have a dual facet. For example, QoL has often been studied as a risk factor but it would exert a protective role when considering the opposite pole, that is, a high QoL. Indeed, many of the factors we study could exert this dual role.

There is a notable discrepancy between studies regarding the relevance of certain protective factors, such as physical and mental health or educational level [65]. It has been proposed that the most important protective factor is having a social network, and that physical and mental health or socioeconomic level hardly play a role [31]. By contrast, physical and cognitive health have been proposed as protective factors for social loneliness but not for emotional loneliness [69]. Beyond the protective factors, fewer studies have focused on the protective and “at risk” groups that all these factors configure. To fill this gap, we analyzed the risk groups using a SPSS “Decision Tree” strategy. By focusing on risk groups and considering the entire population, we found that people with poor mental health, poor QoL and depressive symptoms conform groups at the highest risk of experiencing loneliness. Moreover, half of the people suffering from poor general mental health and depressive symptoms feel lonely. Nevertheless, when there is intermediate mental health but no depressive symptoms and a good QoL, nobody in this group feels lonely. Not suffering depressive symptomatology has previously been considered a protective factor for loneliness [31]. In accordance with our results, among older adults with good mental health, good QoL, and someone caring for them as protective factors, only a few feels lonely (< 2%).

Among people living alone, risk groups are constituted by the same variables as in the overall population, such that older people with poor mental health, poor QoL and depressive symptoms experience more loneliness. By contrast, good mental health and QoL, as well as the perception that someone cares about you, are protective variables that significantly decrease the feeling of loneliness in both the elderly. This last variable is especially relevant because its absence increases perceived loneliness even in the presence of the first two. Similar data has been presented, although there are few studies that have established risk groups. Indeed, it has been proposed that the combination of living alone and having poor health multiplies the probability of feeling lonely by ten when compared to those living with someone and who is having good health [70].

Individuals who do not live alone already have one protective factor and the proportion of those feeling lonely will be lower than among those who do live alone. The “at risk” groups are determined by general mental health and depressive symptoms, as in the case of the participants who live alone, and those in this group are ten times more likely to be lonely than in the population that do not live alone. Older individuals with good mental health and QoL are very unlikely to feel lonely. Once again, these factors prove to be protective variables along with the perception that someone cares about you, a factor that would even outweigh the negative effect of somewhat worse mental health.

Finally, it is worth mentioning that among the predictors of loneliness studied, those with smaller effect sizes do not intervene in the formation of these groups: e.g., educational level, “do not eat hot food more than twice a week”, and “having no one to turn to for help”.

### Latent factors

We identified three latent factors which could correspond to three dimensions of loneliness. We named the first factor the *awareness of psychosocial handicaps*, and the most relevant variable is QoL, followed by general mental health, depressive symptomatology and primary school studies. It is important to emphasize that we do not consider this factor a consequence of loneliness but rather a constituent. The second factor is close to the social dimension of loneliness and it is comprised of three related variables: those who live alone often have no one to turn to for help, and it is more likely that they do not eat properly. All three could be considered variables of deficiency. The second of these factors is an objective reality and the experience of isolation that might be named *objective isolation*. The third factor focuses on the variable “No one is concerned about the older person”, which can be interpreted as a natural conclusion: nobody cares about me and thus, I feel alone. We interpret this factor as the experience of not being of interest or value to anyone and the feeling of having no bonds with others. We refer to this as *subjective or psychological isolation, radical dissociation*. This third factor is close to emotional loneliness in an extreme sense [71] and it also has components of existential loneliness, interpreted as going alone through life, disconnected from others [72].

The multiple dimensions of loneliness are yet to be fully characterized. Different studies using the UCLA [73] or the De Jong Gierveld [74] scales refer to their uni-, bi- or tri-dimensionality [32, 75]. There is some agreement regarding the fundamental dimensions of emotional and social loneliness [4], yet the existence of additional elements as part of the social loneliness dimension has also been suggested, such as the close connection between certain individuals and a wider social network [7]. However, an existential dimension has also been proposed [76]. In short, our results support a three-dimensional construct of loneliness: social, emotional/existential, and “awareness of psychosocial handicaps”.

### Model for the development of loneliness

The factorial study that assessed predictors allows us to distinguish different (groups of) factors related to the feeling of loneliness. Accordingly, we propose a model in which we take into account that causal relationships cannot be established but rather, possible bidirectional associations. The path we propose would not lead to a momentary experience of loneliness but rather, to a protracted and stable experience. However, we must consider alternative paths. Sometimes, loneliness could be triggered by a stressful life event, such as the death of a partner. Alternatively, loneliness can result from a person’s vital trajectory and lifestyle [77].

According to our results, there appear to be objective circumstances related to loneliness, such as living alone, having no one to turn to if help is needed and not eating hot meals (Factor 2 *- Objective isolation*). These circumstances may be associated with limited social relationships and increasing isolation, and the perception of this reality by the older person may induce feelings of loneliness. In addition, the negatively interpreted objective factor and a feeling of being sick produces anxiety, which can derive into insecurity and fear, poor QoL and mental health, and ultimately depressive symptoms (Factor 1 - *Awareness of psychosocial handicaps*). These experiences could increase the risk of objective isolation [78], inducing older people to withdraw from their social environment and not strengthen social ties [79]. They could also be associated with a negatively biased perception of the older person’s current associations, given the influence that unpleasant affective states like anxiety or depression have on the perception of social ties [80], which could strengthen the feelings of loneliness. Finally, the perception and interpretation of worse social relationships (previous or current), or their non-existence, along with the other aforementioned factors, may lead to subjective isolation (a feeling that “nobody cares about me”: Factor 3 - S*ubjective or psychological isolation, radical dissociation*). In conclusion, loneliness could emerge as a result of these proposed interactions and according to this model, links with relevant people and interventions addressing individual factors could halt or minimize these sensations.

### Limitations

This study was not a specific survey on loneliness, although has dealt with broad factors of social kind, health, living habits, food habits and others. The participants were not asked whether they were bothered or distressed by their loneliness, only whether they felt lonely. Furthermore, the study population is drawn from a large city and it may not therefore be representative of other types of the population, like villages or rural environments. Another limitation resides in the type of study and reflects the categorical consideration of loneliness. Loneliness could also be considered as dimensional, although this approach is better suited to non-population clinical studies. This research also has a very important strength, that it is a randomized study such that the sample is representative of the whole population.

## Conclusions

We studied several predictors of loneliness. Living alone, poor mental health (especially depressive symptoms), poor quality of life and living with deficiencies (including having limited educational studies), all form a block of risk factors, and these factors have two polarities. Indeed, they can be protective if we look at their positive side: living with others, good mental health, and good quality of life. Similarly, the feeling that there is someone who cares about oneself, having someone to turn to in case of needing help and having a hot meal (in the sense this has in terms of caring for oneself), or even social support if provided directly by society, are also protective factors. The methodology adopted allowed us to assess these two facets of each factor (risk and protection). Moreover, the model of loneliness development that we outline considers these factors, which although they are individual, they have a significant social component.

Loneliness is now understood as a public health problem and consequently, significant resources are being devoted to reducing or mitigating it. Some strategies can be effective in achieving this goal and among the most widely used at present are those that aim to adapt and enrich the social environment of people who feel lonely. However, we must consider that quality is more important than quantity when considering relationships, and the individual factors associated with this situation are often forgotten.

In addition, protective factors should also be enhanced and health measures that focus on these should be implemented to prevent loneliness before it becomes established, at which point it becomes more challenging to combat.

## Data Availability

The datasets analyzed during the current study are available in the Madrid City Council data repository [https://datos.madrid.es/portal/site/egob].
